# Antidiabetic Effect of an Active Components Group from *Ilex kudingcha* and Its Chemical Composition

**DOI:** 10.1155/2012/423690

**Published:** 2012-03-04

**Authors:** Chengwu Song, Chao Xie, Zhiwen Zhou, Shanggong Yu, Nianbai Fang

**Affiliations:** Key Laboratory of Chinese Medicine Resource and Compound Prescription, Hubei University of Chinese Medicine, Ministry of Education, 1 Huang-jia-hu, Wuhan 430065, China

## Abstract

The leaves of *Ilex kudingcha* are used as an ethnomedicine in the treatment of symptoms related with diabetes mellitus and obesity throughout the centuries in China. The present study investigated the antidiabetic activities of an active components group (ACG) obtained from *Ilex kudingcha* in alloxan-induced type 2 diabetic mice. ACG significantly reduced the elevated levels of serum glycaemic and lipids in type 2 diabetic mice. 3-Hydroxy-3-methylglutaryl coenzyme A reductase and glucokinase were upregulated significantly, while fatty acid synthetase, glucose-6-phosphatase catalytic enzyme was downregulated in diabetic mice after treatment of ACG. These findings clearly provided evidences regarding the antidiabetic potentials of ACG from *Ilex kudingcha*. Using LC-DAD/HR-ESI-TOF-MS, six major components were identified in ACG. They are three dicaffeoylquinic acids that have been reported previously, and three new triterpenoid saponins, which were the first time to be identified in *Ilex kudingcha*. It is reasonable to assume that antidiabetic activity of *Ilex kudingcha* against hyperglycemia resulted from these six major components. Also, synergistic effects among their compounds may exist in the antidiabetic activity of *Ilex kudingcha*.

## 1. Introduction

Diabetes mellitus is one of the most common chronic and systemic diseases in the world. The World Health Organization estimated that diabetes is responsible for approximately 5% of all deaths worldwide and predicted a >50% increase in diabetes-related mortality in 10 years [1]. Dietary restrictions, exercise, and administration of oral glucose-lowering agents are applied widely to control blood glucose concentrations as tightly as possible [2]. Moreover, herbal supplements and other alternative medicines have gradually increased to be used for treatment of diabetic disorders. Kudingcha is the leaves of *Ilex kudingcha *C. J. Tseng (Aquifoliaceae), and a folk medicine for therapy of diabetes in China. Also, the *Ilex kudingcha *have been report to possess antioxidative, hypotensive, antiobesity and antidiabetic activities and contain saponin, polyphenol, and flavones [3–6]. However, there is a lack of reliable data about bioactive components (ACG of *Ilex kudingcha*) for its antiobesity and antidiabetic activities and the effect of ACG on diabetes and obesity.

The present study focused on the effectiveness of ACG on type 2 diabetic mice induced by alloxan. The physiologic and biochemical changes that resulted from ACG treatment were examined. Also, the expression levels of the genes related to glycemia and lipids metabolism were investigated to elucidate the antidiabetic potentials of ACG on type 2 diabetes. Furthermore, a series of LC-DAD/HR-ESI-TOF-MS analyses were carried out to identify the structures of the components present in ACG.

## 2. Materials and Methods

### 2.1. Drugs and Chemicals

The leaves of *Ilex kudingcha*, which grown in Hainan area of China, was obtained from Xianning Kang Jin Chinese Herbal Pieces Co., Ltd. (Hubei, China). The leaves of *Ilex kudingcha* were identified and authenticated by the taxonomist of Key Laboratory of Chinese Medicine Resource and Compound Prescription (Hubei University of Chinese Medicine), Ministry of Education. A voucher specimen (No. 020) was deposited in herbarium of the Key Laboratory.

Alloxan was purchased from Sigma Ltd. (USA). Phenformin was obtained from Merck Sharp pharmaceutical Ltd. (Beijing, China). Cholesterol, triglyceride, blood glucose, superoxide dismutase, malondialdehyde, and nonesterified fatty acid assay kits were purchased from Shanghai Mind Bioengineering Co. Ltd. (Shanghai, China).

### 2.2. Fractionation of the Extract from Ilex Kudingcha

Four kilograms of *Ilex kudingcha *were boiled in 40 L distilled water for 3 h, and this extraction process was repeated for 3 times. The four extracts were combined and concentrated on a rotary evaporator under reduced pressure followed by drying in a freeze dryer. The lyophilized powder of water extract from *Ilex kudingcha *was extracted with 20-fold (w/v) 100% MeOH at 65°C for 3 h and the extraction was repeated for 3 times. The combined 100% MeOH extract was evaporated and dried by a freeze dryer to yield fraction A. The residue was then extracted with 50% MeOH/H_2_O, using same extraction process, to get fraction B. The residue was used as fraction C. The fractions A, B, and C represented 25.4%, 8.4%, and 1.2%, respectively, of the material of *Ilex kudingcha* (w/w). The fractions A, B, and C were stored at −80°C.

### 2.3. Animal Experimental Design

In a previous study, we had compared the antidiabetic effect of the fractions A, B, and C and water extract of *Ilex kudingcha*. The results showed that fractions A and B possess a potent antidiabetic activity on mice with type 2 diabetes induced by alloxan. However, the last residue (fraction C) had no activity under these experimental conditions. In addition, the conclusion from our previous LC-MS data was that the same chemical compositions were present in both fractions A and B, but with different ratios between components in fractions A and B (data not shown). This study focused on the antidiabetic effect of fraction A (ACG).

Male mice (25–30 g) were purchased from Wuhan Institute of Biological Products (Wuhan, China). The mice were housed 8 per cage in a 12 h light/dark cycle at 18–23°C with a humidity of 55–60% for at least 1 week before each study. All animal experimental procedures were approved by the Institutional Animal Care and Use Committee of Hubei University of Chinese Medicine.

Based on the previously established method [7–9], type 2 diabetes was induced by a high-fat diet and alloxan. A high-fat diet contained basic diet (78.8%), egg yolk (10%), lard oil (10%), cholesterol (1%), and cholate (0.2%). The mice were fed this diet for one month, then combined with a twice low-dose fresh alloxan (60 mg*·*Kg^−1^× 2). 48 h after the last alloxan administration, fasting blood was collected from caudal vein of all the animals to determine the glucose concentration. A mouse that had a blood glucose concentration higher than 11 mmol*·*L^−1^ was regarded as type 2 diabetes.

All animals were randomly allocated to one of 6 different 4-week treatments, with 8 mice per group: **C**: control group with 0.4 mL*·*d^−1^ of distilled water; **T**: Type 2 diabetic model group with 0.4 mL*·*d^−1^ of distilled water; **P**: Type 2 diabetic positive group with 50 mg*·*Kg^−1^ *·*d^−1^ phenformine; **K**_**L**_: Type 2 diabetic low-dose-treated group with 1.27 g ACG*·*Kg^−1^ *·*d^−1^; **K**_**M**_: Type 2 diabetic medium-dose-treated group with 2.54 g ACG*·*Kg^−1^ *·*d^−1^; **K**_**H**_: Type 2 diabetic high-dose-treated group with 3.81 g ACG*·*Kg^−1^ *·*d^−1^. The 1.27 g, 2.54 g and 3.81 g ACG were equivalent to 5 g, 10 g, and 15 g *Ilex kudingcha*. The samples (phenformine or ACG powder) were dissolved in 0.4 mL distilled water for intragastric administration. Food and water intake were checked every 4 days.

### 2.4. Collection of Blood and Organ Sample

Blood samples were collected from caudal vein of all the animals every 7 days. After 4 weeks of treatment, all animals were deprived of food for 10 h and given a 2 g*·*Kg^−1^ glucose solution by intragastric administration. Tail blood was collected before the administration of the glucose and 0.5, 1 and 2 h later for oral glucose tolerance test (OGTT). The whole blood was collected by ophthalmectomy after OGTT. The serum was separated by centrifugation at 3500 rpm for 15 min and stored at −80°C until the analysis was carried out. The serum samples were analyzed for cholesterol, triglyceride, blood glucose, superoxide dismutase, malondialdehyde, and nonesterified fatty acid. In addition, liver segments and other organs from each animal were quickly removed and stored at −80°C. The collected liver samples were prepared for total RNA extraction.

### 2.5. Biochemical Analysis

The levels of blood glucose were measured with a commercial kit by a glucose oxidase method. Serum triglyceride and cholesterol concentrations were determined with assay kits by a glycerol-3-phosphate oxidase method and a cholesterol oxidase method, respectively. The levels of serum superoxide dismutase, malondialdehyde, and non-esterified fatty acid were determined by commercial kits according to manufacturer's protocols.

### 2.6. Quantitative Real-Time RT-PCR

Total RNA from mouse liver was extracted using Simply P Total RNA Extraction kit (Bioflux, Japan) according to the manufacturer's instructions and suspended in diethylpyrocarbonate-treated water. The concentration of the total RNA samples was calculated from the optical density by an ultraviolet spectrophotometer set at wavelengths of 260 nm and 280 nm. 

For preparation of cDNA, 2 mg of each total RNA sample was reverse-transcribed using RevertAid First Strand cDNA Synthesis Kit (MBI Fermantas, Vilnius, Lithuania). The resulting cDNA was used to amplify gene-specific cDNAs. Quantitative real-time RT-PCR was performed on a BIO-RAD iCycler machine (CA, USA). A housekeeping transcript, **β**-actin, was used as an internal control because of its stable expression *in vivo* [18]. The primers used for each gene are shown in Table 1. The PCR products were evaluated by their melting curves (data not shown). The amplified gene (4.8 uL) was resolved using agarose gel electrophoresis under 100 V and was stained with GelRed. Analysis of the PCR products was carried out with the Launch Vision Works LS and the Gel Doc-IT Imaging System. The level of mRNA was expressed as the ratio of signal intensity for each gene relative to that of **β**-actin.

### 2.7. LC-DAD/HR-ESI-TOF-MS Analysis

The ACG was thawed at room temperature, dissolved in 80% aqueous methanol (10 mg*·*mL^−1^ of methanol), and used directly for LC-DAD/HR-ESI-TOF-MS analysis. LC-DAD separation was achieved on a 250 × 4.6 mm i.d. Acclaim C18 column (Dionex, USA). Solvent A was water/formic acid (1000 : 1 v/v), and solvent B was acetonitrile/formic acid (1000 : 1 v/v). Solvents were delivered at a total flow rate of 0.5 mL/min. The gradient profile was from 15% B to 35% B linearly in 0–25 min, 35% B to 100% B in 25–45 min and returned to 15% B at 50 min. HR-ESI-TOF-MS analyses were carried out using a MicrOTOF-Q II Focus mass spectrometer (Bruker Daltonics) fitted with an ESI source operating in Auto-MSn mode to obtain fragment ion *m/z*, and internal calibration was achieved with 10 mL of 0.1 M sodium formate solution prior to each chromatographic run. MS operating conditions had been optimized with a capillary of 4500 V (negative ion mode), a capillary of 5000 V (positive ion mode), an end plate offset of −500 V, a collision cell RF of 150 Vpp, a dry heater temperature of 180°C, a dry gas flow rate of 5.0 L/min, and a nebulizer pressure of 3.0 bar. All MS measurements were carried out in the positive and negative ion modes, respectively. 

### 2.8. Statistics

Data were analyzed using GraphPad Prism 5, a computerized statistical analysis program software. The significance of differences between means was evaluated by a *t*-test or one-way analysis of variance. Differences were considered significant at *P* < 0.05. All data are shown as means ± SD.

## 3. Results and Discussion

Most Traditional Chinese Medicine (TCM) remedies are prepared in the form of decoctions and are administered orally. The efficacy of TCM is a characteristic of a complex mixture of chemical compounds which lead to complexity of mechanisms of pharmacological activity. In our previous study, water extract of *Ilex kudingcha* was found to possess an antidiabetic activity with a high does (15 g water extract*·*Kg^−1^ *·*d^−1^ of *Ilex kudingcha*) on mice with type 2 diabetes induced by alloxan. The water extract of *Ilex kudingcha* contained polysaccharides, monosaccharides, proteins, simple organic acids, and other natural products. In addition, the antidiabetic effects of three fractions (A, B, and C) from water extract were examined, and the active components present in *Ilex kudingcha *were low lipophilic chemicals which could be dissolved in hot water or aqueous methanol solvent. Fraction C did not show the antidiabetic effect. The chemicals in Fraction C, which did not dissolved in 50% MeOH/H_2_O solvent, should be polysaccharides, proteins, and other polar chemicals such as monosaccharides and simple organic acids. The purpose of this study was to further clarify the anti-diabetic effect of ACG using alloxan-induced type 2 diabetic mice. Also, the phytochemicals in ACG of *Ilex kudingcha *were systematically analyzed with a DAD-HPLC coupled with online mass spectrometry using an ESI source. 

### 3.1. General Parameters

**Figure 1:**
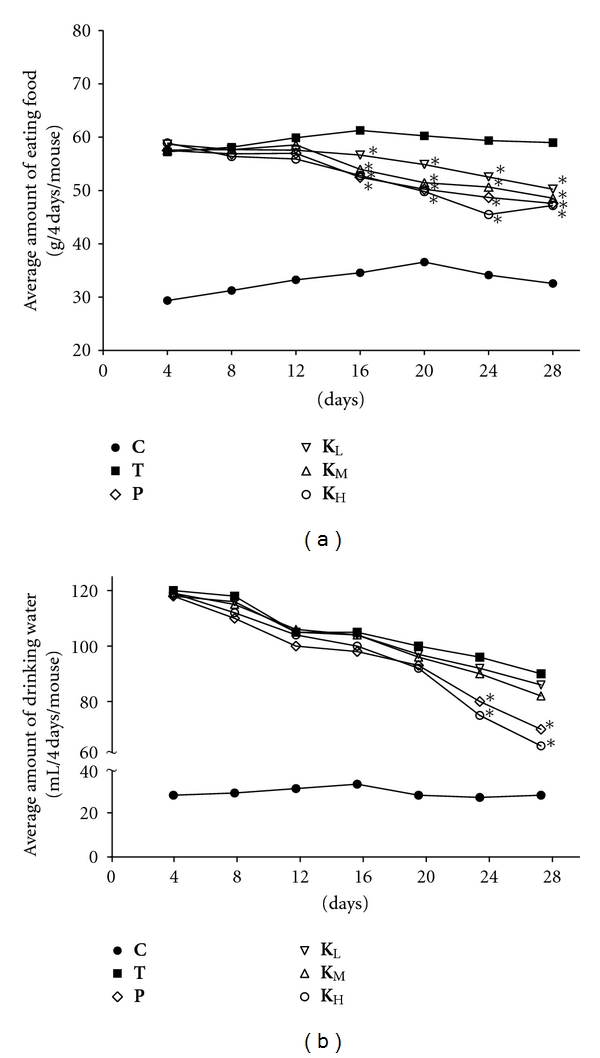
Food (a) and water (b) intake during treatment with ACG in type 2 diabetic mice. *P < 0.05 versus **T** group.

Figure 1 shows the changes in amount of eating food and drinking water during 4 weeks' treatment with ACG. The food intake in **K**_**L**_, **K**_**M**_, and** K**_**H**_ groups was slightly lower than that in **T** group, but the differences were not significant except for the 16–28th days of treatment (*P* < 0.05) (Figure 1(a)). In the 24–28th days, the effect of **K**_**H**_ group was even better than that of **P** group. The water intake of **K**_**H**_ group was significantly lower than that of **T** group (*P* < 0.05) in 24–28th days (Figure 1(b)). As shown in Table 2, the body weight of **K**_**H**_ group was significantly lower than that in **T** groups (*P* < 0.05) after 4 weeks. However, 4 weeks of ACG treatment failed to alter the weight of the liver of type 2 diabetic mice, and there was no difference in the weight of the same organ (heart, kidneys, and pancreas) (data not shown).

Previous studies show alloxan injected to mice resulted in loss of body weight, hyperphagia, and polidypsia. The loss of body weight could be due to dehydration and catabolism of fats and proteins [19]. In the present study, injection of alloxan failed to alter the body weight of mice fed a high-fat diet. And the body weight of mice treated with phenformin have no significant changes compared to that of model mice, suggesting that phenformin may possesses weak weight-losing effectiveness in such a short time. Moreover, treatment with ACG prevented the changes of water intake and food consumption in type 2 diabetic mice. In addition, alloxan-induced diabetes produced a significant increase in glucose levels associated with hyperphagia and polidypsia, which have been decreased by ACG (Figure 1).

### 3.2. Effect of Treatment on the Level of Blood Glucose and OGTT

**Figure 2:**
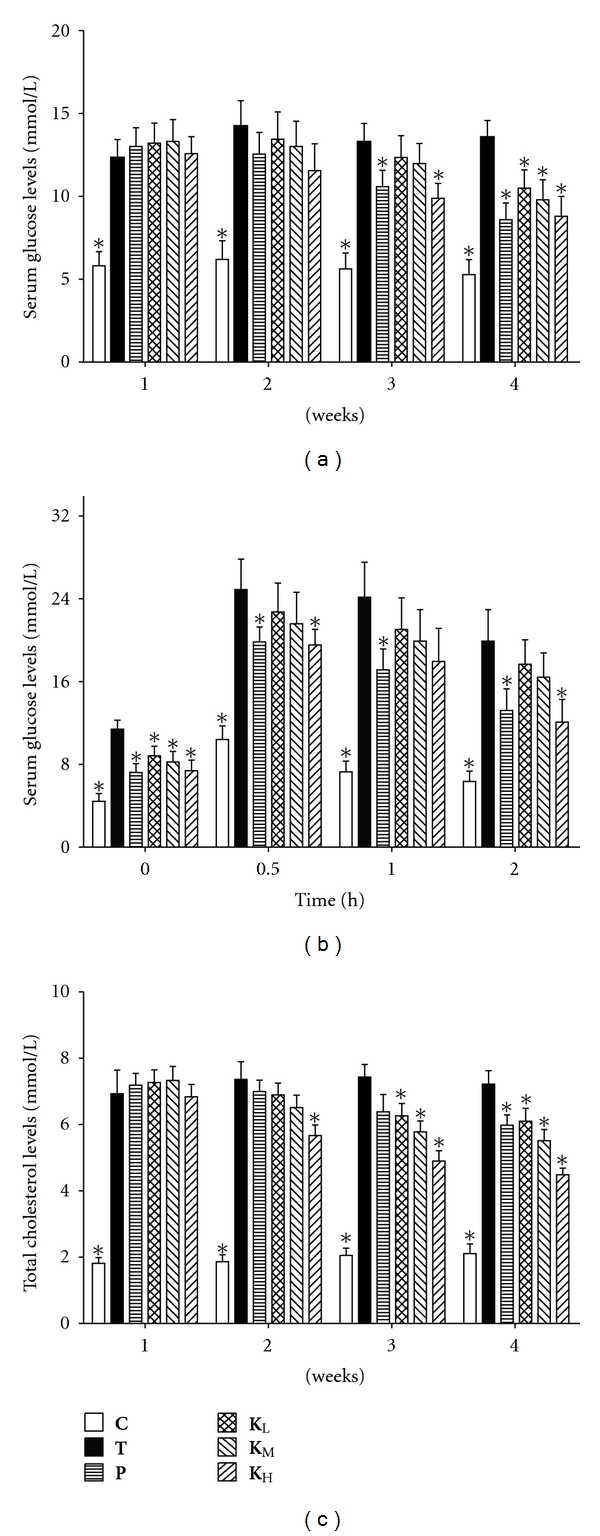
Serum glucose levels (a, b) and total cholesterol levels (c) during treatment with ACG in type 2 diabetic mice.

 Alloxan-induced type 2 diabetic mice had significantly increased concentration of blood glucose compared with mice in **C** group (*P* < 0.05). There was a remarkable decrease in glucose levels of mice treated with ACG for 4 weeks compared to type 2 diabetic mice (*P* < 0.05) (Table 2 and Figure 2(a)). Blood glucose levels increased significantly (*P* < 0.05) after 0.5 h of glucose loading in alloxan-treated type 2 diabetic mice (Figure 2(b)). The blood glucose levels in all groups were elevated after 2 h and they did not recover to the original levels. In addition, the elevation of blood glucose level in **K**_**H**_ group was significantly suppressed in type 2 diabetic mice at 0, 0.5, and 2 h (*P* < 0.05), but was still higher than that of **C** group.

It is found that ACG significantly suppressed the increase in blood glucose levels (Figures 2(a) and 2(b)). Antihyperglycemic effect of ACG observed in alloxan-induced mice can be attributed to several mechanisms. Glucose homeostasis depends largely on the balance between the formation of sugar in the liver and its utilization in liver, muscle, and adipose tissue. Therefore, energy metabolism in the other organs such as adipose tissue and skeletal muscle might be importantly related to the glucose metabolism of the ACG groups, leading to the amelioration of glucose metabolism.

### 3.3. Serum Lipid Measurements

Table 2 and Figure 2(c) show that cholesterol concentrations in serum were significantly increased in **T** group (*P* < 0.05) when compared with nondiabetic mice in **C** group. However, the alteration in lipid metabolism was partially attenuated as evidenced by decreased serum cholesterol levels after treatment with ACG. Meanwhile, the serum triglyceride levels slightly decreased in **K**_**L**_, **K**_**M**_, and **K**_**H**_ groups. In addition, nonesterified fatty acid levels were lower after 4 weeks in **K**_**L**_, **K**_**M**_, and **K**_**H**_ groups when compared with diabetic controls, but they did not recover to normal level.

Diabetes is associated with profound alterations in the plasma lipid and lipoprotein profile as well an increased risk of coronary heart disease [20]. In the present study, the ability of ACG to partially reverse the hyperglycemia of alloxan-treated mice was confirmed. In addition to the hypoglycemic activity of ACG, it also possessed a potent lipid lowering properties in type 2 diabetic mice. The levels of serum cholesterol, triglyceride, and nonesterified fatty acid in alloxan-induced diabetes were higher than that of **C** group. ACG treatment ameliorated these effects, possibly by controlling the hydrolysis of certain lipoproteins and their selective uptake and metabolism by different tissues.

### 3.4. Antioxidant Activity of ACG

The activities of superoxide dismutase and malondialdehyde concentration in serum of mouse are shown in Table 2. The activities of superoxide dismutase were suppressed in alloxan-induced type 2 diabetic mice. Furthermore, induction of diabetes by alloxan caused a marked rise in serum malondialdehyde. However, a significant reactivation of antioxidant enzymes was observed in **K**_**H**_ group (*P* < 0.05) after treatment with 3.81 g ACG*·*Kg^−1^ *·*d^−1^. The levels of serum malondialdehyde were reversed in **K**_**L**_, **K**_**M**_, and **K**_**H**_ groups as compared to that of **T** group, but the changes were not significant. In addition, mice treated with phenformin showed an increased superoxide dismutase level and decreased malondialdehyde level compared to that of model mice. The effectiveness was reported previously [21].

There is an association between oxidative stress and diabetes particularly through the generation of lipid peroxidation. It is known that hyperglycemia can result in the generation of reactive oxygen species and that it also inhibits the activity of antioxidant enzymes by glycosylation [22]. Superoxide dismutase is a metalloenzyme which involved in the dismutation of the superoxide anion to molecular oxygen and hydrogen peroxide [23]. It is reported that diabetics usually exhibit high oxidative stress due to persistent and chronic hyperglycemia, which thereby depletes the activity of antioxidative defense system and thus promotes free radicals generation. And as byproduct of lipid peroxidation, malondialdehyde concentration reflects the degree of oxidation in the diabetic mice [24]. In the present study, superoxide dismutase and malondialdehyde were examined to find out the possible mechanism involved in the observed results. The results show that ACG might affect lipid profile and are responsible for their antidiabetic properties by slightly reducing serum malondialdehyde level and improving serum superoxide dismutase activity to attenuate the lipid peroxidation caused by various forms of free radicals.

### 3.5. Quantitative Real-Time RT-PCR

**Figure 3:**
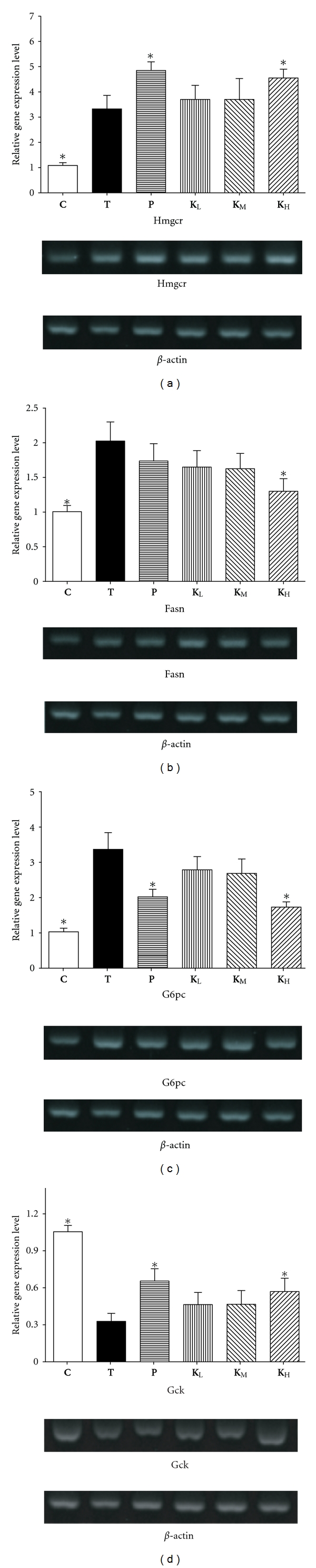
Gene expression analysis in the liver by real-time RT-PCR. (a) HMGCR; (b) FASN; (c) G6PC; (d) GCK. Significant differences were observed at *P* < 0.05* versus **T** groups. **β**-actin was used as a control to standardize the efficiency of each reaction. Gene expression was presented using a modification of the 2^−ΔΔCt^ method [12–14].

**Figure 4:**
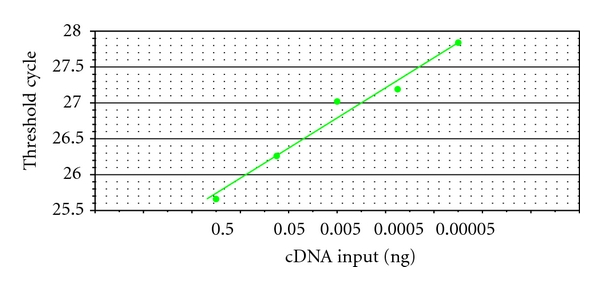
Fold-change on RT-PCR. The corresponding RT-PCR efficiencies were calculated according to the equation *E* = 10^[−1/slope]^ [12].

To validate the biochemical changes, four genes were examined by real-time RT-PCR. The representative genes were selected according to metabolic functions in terms of gluconeogenesis (G6pc), glycolysis (Gck), lipid metabolism (Fasn), and cholesterol synthesis (Hmgcr). As shown in Figure 3, the expressions of Gck and Hmgcr in** K**_**H**_ group were significantly higher than that in **T** group on RT-PCR evaluation. The expressions of G6pc and Fasn in **K**_**H**_ group was significantly lower than that in **T** group.  Figure 4 shows that the results of real-time RT-PCR correlated well (*E* = −98.7%, *R* ^2^ = 0.975, slope = 0.527, *y*-int = 27.849), indicating that gene expression profile by RT-PCR was highly reliable, and housekeeping transcript **β**-actin was suitable to standardize the efficiency of each reaction.

Gene expression of Hmgcr, a key enzyme in cholesterol synthesis pathway, was upregulated significantly in** K**_**H**_ group indicating that cholesterol synthesis is potently induced by ACG treatment. In those diabetic mice, ingestion of ACG increased the activity of Hmgcr which resulted in the lowering of plasma cholesterol levels. These results may be attributed to the increased excretion of bile acid and cholesterol. The ACG treatment induced a deficiency in hepatic cholesterol and its derivatives, leading to the potent induction of cholesterol synthesis and cholesterol uptake in the liver [25]. ACG ingestion upregulated the expression of Gck gene and downregulated the expression of G6pc gene related to glycometabolism (Figures 3(c) and 3(d)). Gck is a member of the hexokinase family (hexokinase type IV) that catalyzes the first committed step in glycolysis. Either upregulation of Gck gene expression or Gck enzyme activity has been reported to be able to suppress an increase in blood glucose [26]. These indicate that ACG ingestion activates glycolysis. Another downregulated gluconeogenesis-related gene, G6pc, is a key enzyme in gluconeogenesis, catalyzing the hydrolysis of D-glucose 6-phosphate to D-glucose. Thus, we concluded that ACG ingestion-induced suppression of the increase in blood glucose levels was attributed mainly to the activation of glycolysis and inactivation of gluconeogenesis in liver.

### 3.6. LC-DAD/HR-ESI-TOF-MS Analysis

In this study, sixteen components including three chlorogenic acids isomers, three dicaffeoylquinic acids isomers, four flavonoids, and six triterpenoid saponins were identified or characterized by their MS/MS spectra and LC retention time.

Compounds **1**(HR-ESI-MS: *m/z* 353.0953 [M-H]^−^), **2** (HR-ESI-MS: *m/z* 353.0950 [M-H]^−^), and **3 **(HR-ESI-MS: *m/z* 353.0957 [M-H]^−^) had the same [M-H]^−^ ion in accordance with a C_16_H_17_O_9_ formula of chlorogenic acid (calculated for C_16_H_18_O_9_, 354.0951). Their product ions at *m/z* 135 and 191 from collision-induced dissociation (CID) indicated that these three compounds were chlorogenic acids isomers (Figure 6). The three chlorogenic acids isomers neochlorogenic acid, chlorogenic acid, and cryptochlorogenic acid have been identified in dried plums and *Ilex kudingcha* by LC-MS/MS [10, 11]. By comparison of the peak areas and retention time of these three chlorogenic acids isomers on the C18 HPLC column, compounds **1**, **2**, and **3** were identified here as neochlorogenic acid, chlorogenic acid, and cryptochlorogenic acid, respectively. Compounds **8**, **9,** and **10** (HR-ESI-MS: [M-H]^−^ at *m/z* 515.1269 for **8**, 515.1270 for **9** and 515.1262 for **10**) had the same [M-H]^−^ ion (Figure 7) in accordance with a C_25_H_23_O_12_ formula of dicaffeoylquinic acid (calculated for C_25_H_24_O_12_, 516.1267), and their product ions at *m/z* 179, 135, 173, and 191 from CID, indicated that these compounds might be the dicaffeoylquinic acids isomers. These compounds has been reported in *Ilex kudingcha* previously [10]. Four pairs of ions: [M-**A_1_**]^−^and **A_1_**; [M-**A_2_**]^−^and **A_2_**; [M-**B_1_**]^−^and **B_1_**; and [M-**B_2_**]^−^ and **B_2_** in MS/MS from negative ion mode of the [M-H]^−^ ions at *m/z* 353 (compounds **1**, **2**, and **3**) and 515 of (compounds **8**, **9**, and **10**) (Table 3) suggested the diagnostic fragmentation patterns of chlorogenic acid isomers and dicaffeoylquinic acid isomers. The diagnostic fragmentation patterns involved cleavage of intact caffeoyl and quinic acid fragments [11]. Using ESI-MS/MS in the positive ion mode, the protonated molecular ions of chlorogenic acid isomers and dicaffeoylquinic acid isomers gave only one ion at *m/z* 163. The typical fragmentation pathway resulted from the positive ionization of the carbonyl oxygen [11]. In addition, MS/MS of the [M-H]^−^ ion at *m/z* 707 appeared on the side of peak** 2** and it was identified as a dimeric adduct ion of chlorogenic in previous report [10].

HR-ESI-MS of compound **4** exhibited a deprotonated molecular ion [M-H]^−^at *m/z* 609.1536 corresponding to C_27_H_29_O_16_ (calculated for C_27_H_30_O_16_ [M-H]^−^, 610.1533), and this compound was identified as rutin (quercetin 3-rutinoside) based on product ions from CID of [M-H-146]^−^ at *m/z* 463 and [M-H-146–162]^−^ at *m/z* 301, as reported previously [10]. Based on the ESI MS/MS data, CID pathways of compounds **5** and **6 **were similar to that of compounds** 4**, which suggest that these compounds possessed a same aglycon quercetin with different glycosides. Compound **5** had the [M-H]^−^ ion at *m/z* 463 and product ion at *m/z* 301 [M-H-162]^−^ from CID of [M-H]^−^, which suggested that compound **5** was quercetin 3-glucoside. Compound **6** had the [M-H]^−^ at *m/z* 595 with its product ions [M-H-132-162]^−^ at *m/z* 301 and [M-H-132]^−^ at *m/z* 463 suggested that compound 6 was quercetin 3-vicianoside. For the compound **7**, the [M-H]^−^ at *m/z* 593 corresponding to C_27_H_29_O_15_, with its product ions [M-H-146–162]^−^ at *m/z* 285 and [M-H-146]^−^ at *m/z* 447 suggested that compound **7** was kaempferol 3-rutinoside. This compound has been reported in* Ilex kudingcha *[10].

Compound **11 **had the [M-H]^−^ion at *m/z* 745.4247 (calculated for C_41_H_62_O_12_, 746.4241) was identified as 3-*O*-*α*-L rhamnopyranosyl-(1-2)-*α*-L-arabinopy-ranosyl-*α*-kudinlactone based on product ions from CID of [M-146]^−^ and [M-H-146-132]^−^ (Table 4 and Figure 8), as reported in *Ilex kudingcha* previously [15]. HR-ESI-MS of compound **12 **exhibited an [M-H]^−^ ion at *m/z* 1073.5695 corresponding to C_53_H_85_O_22_ (calculated for C_53_H_86_O_22_, 1074.5611), and four ions at [M-H-162]^−^, [M-H-162-162]^−^, [M-H-162-162-146]^−^, and [M-H-162-162-146-132]^−^ indicated four sugars in the structure. It showed similar CID fragmentation with macranthoside B [16]. The MS/MS data from **13** were almost identical to those of **12 **(Table 4), and it was likely that **13 **is a stereoisomer of **12 **due to different configuration of the triterpene ester. Compound **15** exhibited an [M-H]^−^ ion at *m/z* 971.4936 corresponding to C_48_H_75_O_20_ (calculated for C_48_H_76_O_20_ [M-H]^−^, 972.4929). Its MS/MS spectrum gave two ions of [M-H-162]^−^ and [M-H-162-162]^−^, strongly suggesting the presence of two sugar moieties. HR-ESI-MS of compound **16 **displayed the [M-H]^−^ ion at *m/z* 955.4991 corresponding to C_48_H_75_O_19_ (calculated for C_48_H_76_O_19_ [M-H]^−^, 956.4981). Like compound **15**, the loss of 162 Da and 324 Da originated from the glucoside unit. Based on the high intensity signals, compound** 15** was identified as 3-O-*β*-D-glucopyranosyl-(1-4)-**β**-D-glucuronopyranosyl siaresinolic acid-28-O-**β**-D-glucopyranosyl ester and compound **17 **was identified as 3-O-**β**-D-glucopyranosyl-(1-4)-**β**-D-glucuronopyranosyl oleanolic acid-28-O-**β**-D-glucopyranosyl ester. These two compounds have been reported in* Ilex godajam *and* Ilex hylonoma *[17]. In addition, compound **16 **was characterized as isomers of compound **17** due to the same [M-H]^−^ ions and fragmentation patterns. The compounds **5**, **6**, **12**, **13**, **15**, **16**, and **17** were newly found in *Ilex Kudingcha*. Since NMR data and the corresponding standards of the compounds **13** and **16** were not available, identifications of these compounds could not be completed by the LC-MS/MS in this study.

**Figure 5:**
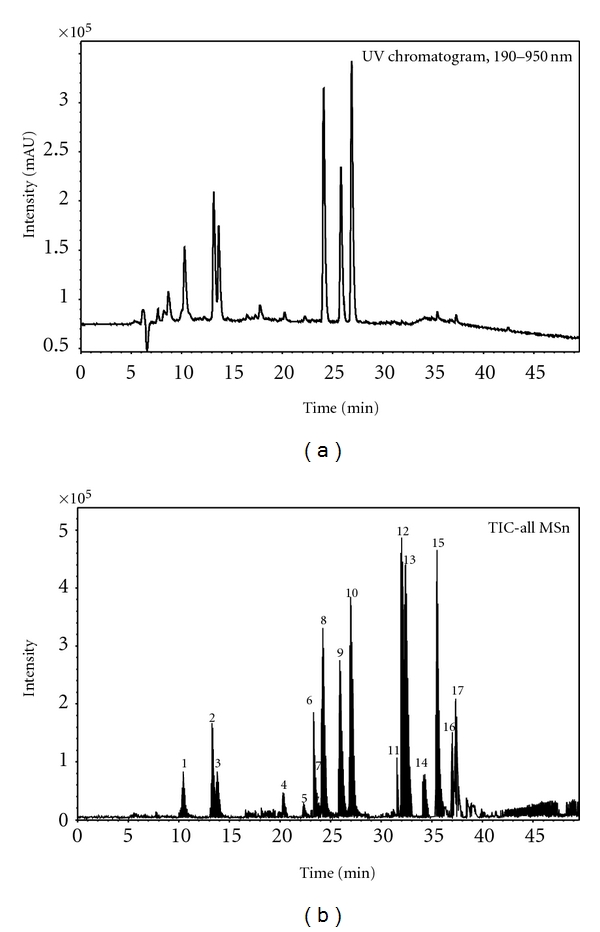
LC-DAD chromatogram spectrum and HR-ESI-TOF-MSn negative mass spectrum of ACG.

**Figure 6:**
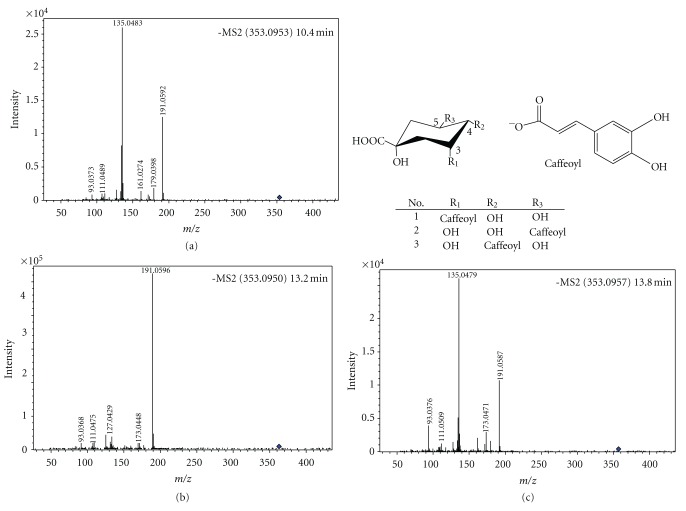
HR-ESI-TOF-MS/MS of three chlorogenic acids isomers identified in ACG and their structures. (a) Spectrum of Compound **1**; (b) spectrum of Compound **2**; (c) spectrum of Compound **3**.

**Figure 7:**
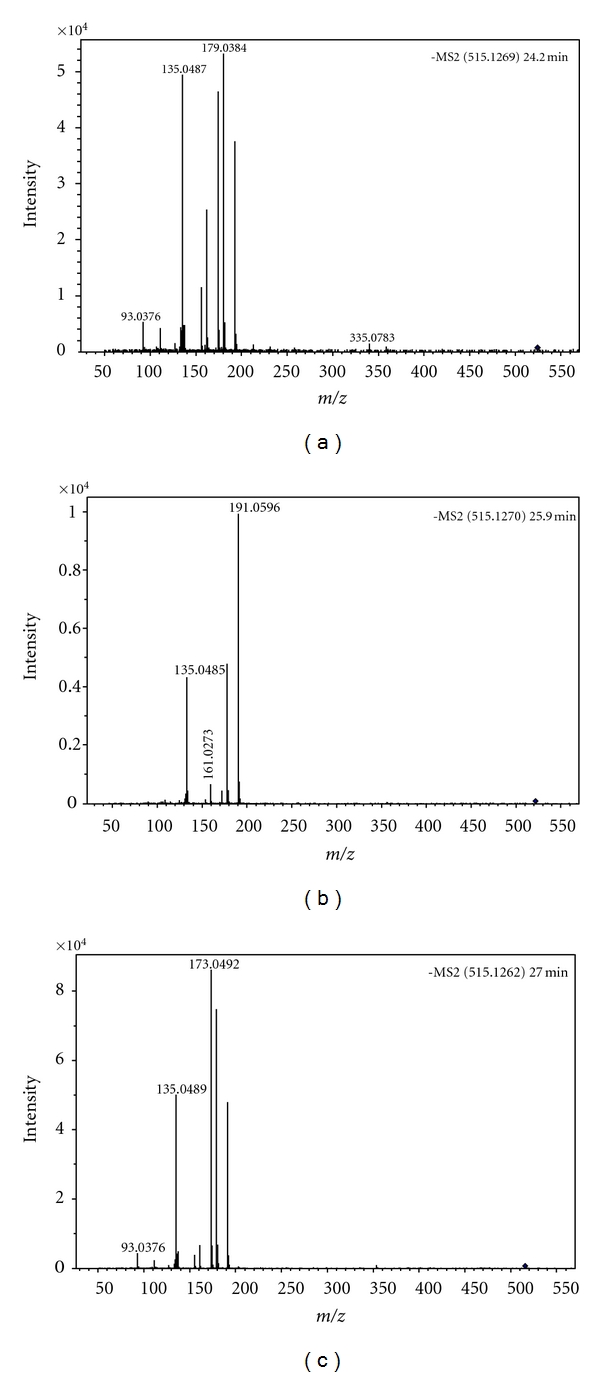
HR-ESI-TOF-MS/MS of three dicaffeoylquinic acid isomers identified in ACG. (a) Spectrum of Compound **8**; (b) spectrum of Compound **9**; (c) spectrum of Compound **10**.

**Figure 8:**
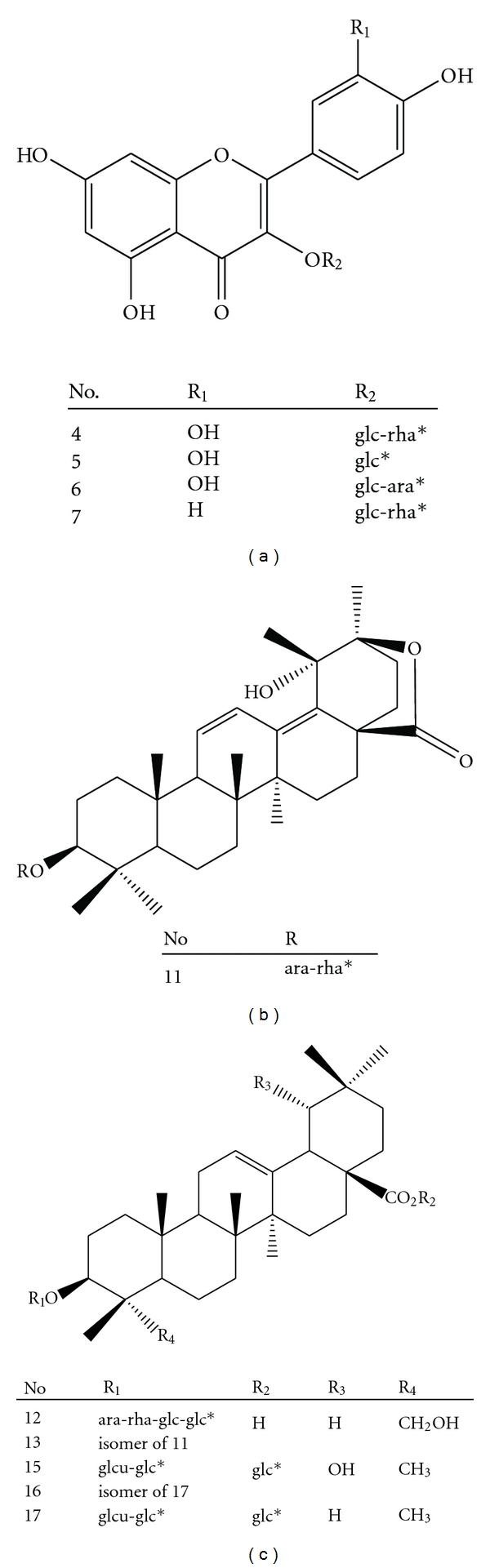
Structures of the flavonoids and triterpenoid saponins identified or characterized in ACG. *ara: arabinoside; rha: rhamnoside; glc: glucoside; glcu: glucuronide.

The flavonoids have long been recognized to possess anti-inflammatory, antioxidant, antiallergic, antiatherosclerotic and antidiabetic effects [27]. Previous study showed that rutin possessed partial protective effect on multiple low-dose streptozotocin-induced diabetes in mice [28]. However, rutin and its similar compounds orally administered to diabetic mice should not decrease elevated blood glucose level in short-time (4 weeks) [29]. In addition, dicaffeoylquinic acids have attracted attention because of its potentiating effect on bile secretion and, therefore, its moderating effect on blood cholesterol levels [11]. Also, dicaffeoylquinic acids have been studied as a potentially important class of HIV inhibitors that act at a site distinct from that of current HIV therapeutic agents [30]. It also has been reported that *Ilex kudingcha* total saponins could improve total cholesterol, triglyceride in ApoE^−/−^ mice [31]. While flavonoids and chlorogenic acids have a strong UV absorption, small UV peaks of **1**–**7** indicated that flavonoids (**4–7**) are trace components and chlorogenic acids (**1**, **2**, and **3**) are minor components in ACG (Figure 5). Therefore, the major principles in ACG are three dicaffeoylquinic acids (**8**, **9**, and **10**) and three triterpenoid saponins (**12**, **13**, and **15**).

## 4. Conclusions

ACG treatment significantly reduced the elevated levels of serum glycaemic and lipids in type 2 diabetic mice and improved their levels of genes those related to type 2 diabetes. It is reasonable to assume that antidiabetic activity of *Ilex kudingcha* against hyperglycemia is resulted from the major principles including three dicaffeoylquinic acids and three triterpenoid saponins. Also, it is possible that synergistic effects among their compounds exist in the antidiabetic activity of *Ilex kudingcha*.

## Figures and Tables

**Table 1:** Polymerase chain reaction primer sequences.

Mouse gene	Primer	Sequence**
*β*-Actin	Forward Reverse	CAC TgT gCC CAT CTA CgA CAg gAT TCC ATA CCC AAg
Fasn*	Forward Reverse	Aag Cgg CCA TTT CCA TTg CgT ACC Tgg ACA Agg ACT TTg
G6pc*	Forward Reverse	AAT CTC CTC Tgg gTg gCA gCT gTA gTA gTC ggT gTC C
Hmgcr*	Forward Reverse	gTT CTT TCC gTg CTg TgT TCT ggA CTg ATA TCT TTA gTg CAg AgT gTg gCAC
Gck*	Forward Reverse	CCC TgT Aag gCA CgA AgA Cgg Aga AgT CCC Acg Atg T

*Fasn: fatty acid synthetase; G6pc: glucose-6-phosphatase catalytic enzyme; Hmgcr: 3-hydroxy-3-methylglutaryl coenzyme A reductase; Gck: glucokinase.

**Primers are shown 5′→3′.

**Table 2:** Physiological and Biochemical parameters of mice in various groups after 4-week treatment of ACG (mean ± SD, *n* = 8).

	**C**	**T**	**P**	**K**_**L**_	**K**_**M**_	**K**_**H**_
BW** (g)	30.96 ± 1.45*	38.53 ± 1.87	36.45 ± 3.56	35.47 ± 3.68	34.29 ± 3.81	32.58 ± 2.04*
GLU** (mmol/L)	5.28 ± 0.90*	13.56 ± 1.02	8.60 ± 1.01*	10.50 ± 1.10*	9.80 ± 1.21*	8.81 ± 1.02*
TC** (mmol/L)	2.30 ± 0.32*	7.90 ± 0.44	6.54 ± 0.34*	6.67 ± 0.43*	6.03 ± 0.37*	4.90 ± 0.22*
TG** (mmol/L)	1.03 ± 0.42	1.38 ± 0.47	1.28 ± 0.31	1.34 ± 0.33	1.29 ± 0.58	0.98 ± 0.13*
SOD** (U/mL)	248.45 ± 49.56*	135.90 ± 46.72	227.67 ± 52.40	205.34 ± 43.50	224.91 ± 64.30	256.46 ± 56.47*
MDA** (nmol/mL)	6.24 ± 1.30*	16.74 ± 3.65	12.08 ± 6.09	14.45 ± 3.21	14.31 ± 4.65	13.24 ± 3.81
NEFA** (mmol/L)	1.89 ± 0.19*	3.75 ± 0.44	2.43 ± 0.15*	3.04 ± 0.24	2.97 ± 0.47	2.19 ± 0.21*

*P < 0.05 significantly different from **T** group.

**BW: body weight; TC: cholesterol; TG: triglyceride; GLU: serum glucose; SOD: superoxide dismutase; MDA: malondialdehyde; NEFA: nonesterified fatty acid.

**Table 3:** HR-ESI-TOF-MSn data of major phenolic compounds identified in ACG.

		CID spectra of [M-H]^−^ (relative intensity, %)					CID spectra of [M+H]^+^		
Peak no.*	*R_t_* min	[M-H]^−^	pathway 1			pathway 2	(relative intensity, %)		
			B_1_**	B_2_**	A_2_**	A_1_**	[M+H]^+^	B_3_**	Lit. report

chlorogenic acids isomers									
**1**	10.4	353 (0.1)	179 (7.0)	135 (100)		191 (48.2)	355 (0.1)	163 (100)	[10, 11]
**2**	13.2	353 (0.3)	179 (1.8)	135 (6.7)	173 (2.9)	191 (100)	355 (0.1)	163 (100)	[10, 11]
**3**	13.8	353 (0.4)	179 (6.0)	135 (100)	173 (11.1)	191 (41.1)	355 (0.1)	163 (100)	[10, 11]

dicaffeoylquinic acids isomers									

**8**	24.2	515 (0.1)	179 (100)	135 (92.9)	173 (87.2)	191 (70.4)	517 (0.1)	163 (100)	[10, 11]
**9**	25.9	515 (0.1)	179 (48.8)	135 (43.5)	173 (4.3)	191 (100)	517 (0.1)	163 (100)	[10, 11]
**10**	27.0	515 (0.1)	179 (86.9)	135 (58.2)	173 (100)	191 (55.6)	517 (0.1)	163 (100)	[10, 11]

*The numbers of the peaks in this table coincide with the numbers of the peaks in Figure 5.

**The definitions of B1, B2, B3, A1, and A2 were described in [11].

**Table 4:** HR-ESI-TOF-MSn data of flavonoids and triterpenoid saponins identified in ACG.

peak no.*	*R_t_* min	CID spectra of [M-H]^−^ (relative intensity, %)			Identification	Lit. report
		[M-H]^−^	Base ion	Other ions		

Flavonoids						
**4**	20.3	609 (1.8)	301 (100)	463 (9.0), 271 (22.2)	Quercetin 3-rutinoside	[10]
**5**	22.4	463 (1.8)	301 (100)	285 (6.2), 271 (77.7)	Quercetin 3-glucoside	
6**	23.0	595 (4.6)	301 (100)	463 (24.7), 285 (8.4)	Quercetin 3-vicianoside	
7**	23.7	593 (4.3)	285 (100)	447 (23.8), 255 (37.6)	Kaempferol 3-rutinoside	[10]

Triterpenoid saponins						

**11**	31.5	745 (1.1)	467 (100)	599 (48.3), 369 (14.6)	3-*O*-*α*-L-Rhamnopyranosyl-(1-2)-*α*-L-arabinopy-ranosyl-*α*-kudinlactone	[15]
**12**	32.0	1073 (0.1)	749 (100)	911 (19.8), 603 (7.9), 471 (3.6)	Macranthoside B	[16]
**13**	32.4	1073 (0.2)	749 (100)	911 (12.8), 603 (8.0), 471 (7.8)	Isomer of 12	***
**15**	35.5	971 (0.1)	809 (100)	763 (49.9), 647 (6.5), 471 (8.9)	3-O-*β*-D-Glucopyranosyl-(1-4)-*β*-D-Glucuronopyranosyl siaresinolic acid-28-O-*β*-D-glucopyranosyl ester	[17]
**16**	37.0	955 (0.3)	793 (100)	631 (42.6), 455 (8.5)	Isomer of 17	***
**17**	37.3	955 (0.8)	793 (100)	631 (38.6), 455 (10.2)	3-O-*β*-D-Glucopyranosyl-(1-4)-*β*-D-glucuronopyranosyl oleanolic acid-28-O-*β*-D-glucopyranosyl ester	[17]

Unknown compound						

**14**	34.1	582 (0.6)	374 (100)		Unknown	

*The numbers of the peaks in this table coincide with the numbers of the peaks in Figure 5.

**Compounds **6** and **7** were not clearly separated in LC-MS analysis.

***Since NMR data and the corresponding standards of these compounds were not available, identification of these compounds could not be completed by the LC-MS/MS in this study.
